# RISK FACTORS OF OSTEOARTHRITIS AMONG OLDER ADULTS WITH COGNITIVE DECLINE IN SOUTH KOREA

**DOI:** 10.1093/geroni/igad104.2954

**Published:** 2023-12-21

**Authors:** Jennifer (Jeehyun) Kim, Bada Kang

**Affiliations:** Yonsei University, College of Nursing, Seoul, Republic of Korea; Yonsei University, Seoul, Republic of Korea

## Abstract

Individuals with cognitive decline are vulnerable to developing osteoarthritis owing to reduced physical activity and impaired communicational ability. While the cross-sectional relationship between cognitive decline and chronic diseases has been previously studied, longitudinal studies are needed to identify determinants associated with the onset of osteoarthritis among older adults with cognitive decline. This study was aimed at elucidating the risk factors of osteoarthritis based on cognitive status. 1,142 older adults, including 334 cognitive decline and 808 normal cognition individuals, were selected from the Korean Longitudinal Study of Aging. Kaplan-Meier curves were plotted to estimate the probability of osteoarthritis based on the participants’ sex and cognitive status. Cox proportional hazard regression analysis was performed to determine factors associated with the risk of osteoarthritis after the participants were stratified by cognitive status. Female sex and advanced age were associated with a greater risk of osteoarthritis in both the participants with and without cognitive decline. Furthermore, the risk of osteoarthritis was significant among older adults with cognitive decline if they lived alone, were depressed, had social engagement fewer than once per week, or had a high dependency on others for completing activities of daily living. However, there was an increased risk of osteoarthritis among older adults with normal cognition who had a high body mass index and a chronic medical condition. Therefore, comprehensive coping strategies focusing on the socio-environmental characteristics and clinical symptoms of older adults with cognitive decline should be considered to prevent a further increase in the healthcare burden associated with osteoarthritis.

